# Evaluation of the Potential Anti-Inflammatory Activity of Black Rice in the Framework of Celiac Disease

**DOI:** 10.3390/foods12010063

**Published:** 2022-12-22

**Authors:** Stefano Piazza, Francesca Colombo, Corinne Bani, Marco Fumagalli, Olimpia Vincentini, Enrico Sangiovanni, Giulia Martinelli, Simone Biella, Marco Silano, Patrizia Restani, Mario Dell’Agli, Chiara Di Lorenzo

**Affiliations:** 1 Department of Pharmacological and Biomolecular Sciences (DiSFeB), Università degli Studi di Milano, 20133 Milan, Italy; 2 Unit of Human Nutrition and Health, Department of Food Safety, Nutrition and Veterinary Public Health, Istituto Superiore di Sanità, 00161 Roma, Italy; 3 Coordinating Research Center (CRC) “Innovation for Well-Being and Environment”, Università degli Studi di Milano, 20122 Milan, Italy

**Keywords:** celiac disease, black rice, phenolic compounds, antioxidant activity, anti-inflammatory activity, gliadin, in vitro digestion

## Abstract

Inflammation and oxidative stress are two mechanisms involved in the pathogenesis of celiac disease (CD). Since the direct effect of gliadin on the intestinal epithelia is less studied, the aims of this study were the development of a specific cellular model based on the use of gliadin as a pro-inflammatory stimulus and the evaluation of the potential antioxidant and anti-inflammatory properties of extracts from different black rice in the framework of CD. The rice extracts were in vitro digested, characterized in terms of phenolic compounds and antioxidant capacity, and tested on Caco-2 cells to investigate their inhibitory effect on Reactive Oxygen Species, the NF-κB transcription and the CXC chemokines (sICAM-1, IL-8, and CXCL-10). In addition, the role of the extracts in modulating the activation of epithelial cells in CD was confirmed by applying the K562(S) agglutination test. The black rice extracts showed inhibitory effects on the production of the oxidative and the inflammatory mediators considered, with particular reference to lymphocyte-attracting CXCL-10 both before and after digestion. The presence of anthocyanins and their digestion metabolites may account for the observed anti-inflammatory activity after in vitro digestion. This work provided preliminary data supporting the use of black rice as a healthy food or ingredient of food supplements for celiacs.

## 1. Introduction

Celiac disease (CD) is an autoimmune, inflammatory disorder triggered in individuals with a genetic predisposition associated with the ingestion of toxic prolamins (wheat gliadin, rye secalin, and barley hordein). Celiac disease is a worldwide health problem with a global prevalence of 1.4%, based on serologic tests, and of 0.7%, based on biopsy [1]. In celiac disease, toxic prolamins can activate the innate ad adaptive immune response leading to a duodenal mucosal atrophy. Some gliadin regions are responsible for cytotoxic or immunomodulatory activities, while other sequences trigger off the oxidative stress at intestinal level and induce the release of proinflammatory cytokines [2]. In analogy with other autoimmune diseases, the intestinal mucosa of CD patients shows increased levels of IFN-γ and CXC chemokines (such as CXCL-8 and CXCL-10), involved in the amplification of gluten-specific T cells response (for a deeper description of CD pathogenesis, see the review from Ramírez-Sánchez et al. [3]). IFN-γ and innate cytokines (such as IL-1β) are well-known synergistic effectors causing epithelial damage through the activation of inflammatory and apoptotic pathways, such as JAK/STAT and NF-κB [4]. Therefore, inflammation and oxidative stress seem to be involved in the pathogenesis of CD [5,6,7], and the gluten-free diet in celiac patients improves the functionality of the intestinal mucosa only partially [8,9]. Beyond the role as immunological hapten, gliadin has been rarely considered for its direct effect on intestinal mucosa. Capozzi et al. observed that the treatment of Caco-2 cells with digested gliadin determines the secretion of two key pro-inflammatory cytokines (i.e., IL-6 and IL-8) and the phosphorylation of IRAK1 with the consequent NF-κB activation [10]. Similar results were reported by Gupta et al. who observed that gliadin proteins tested on human intestinal cell lines (HCT-116 and HT-29) increased the mRNA levels of different cytokines (such as IL-1 β, IL-6, IL-10, IL-15, IL-18) and the mRNA levels of IFN-γ and TNF-α genes [11,12]. The potential beneficial role as anti-inflammatory agents of some phytochemicals or botanicals (i.e., curcumin and green tea extracts), evaluated in the framework of CD, underlined the ability of natural compounds to reduce in vitro cytokine expression [12,13]. Therefore, the consumption of foods rich in dietary antioxidants could mitigate the oxidative stress and the inflammatory status characteristic of celiac patients, thus improving their health. Rice is naturally gluten-free, and it is one of the most consumed cereals among celiac subjects, both as raw rice [14] and flour, often used as ingredients of gluten-free products [15]. The pigmented varieties of cereals are richer in bioactive compounds (such as anthocyanins and other flavonoids) and show higher antioxidant activity compared to the non-pigmented ones. In fact, as reported in the study by Shen et al., 2009 [16], the total antioxidant capacity measured using the ABTS test can vary widely when different varieties of rice are considered (white, red, and black rice). Regarding white rice, the average ABTS was 0.196 mM TEAC, while red rice had an average of 1.705 mM TEAC. Black rice samples had an average antioxidant capacity of 4.484 mM TEAC, about three times that of red rice.

On these bases, pigmented brown rice is recognized as a useful functional food with potential beneficial and nutritional properties thanks to its high content of bioactive compounds, including anthocyanins and other phenolic compounds.

Several in vitro studies have reported the potential beneficial effects of pigmented rice, such as anti-inflammatory and diuretic activity [17,18]. Furthermore, in the study by Tanaka et al. [19], purple rice extract (*Oryza sativa* L.) and relative compounds showed in vitro an inhibition of VEGF-induced angiogenesis. Therefore, pigmented cereals could represent potential interesting ingredients for the formulation of functional cereal-based products [20]. The consumption of pigmented rice could contribute to improve the health not only of the general population, but also of people suffering from chronic diseases involving the gastrointestinal system, such as celiac consumers.

The aim of this study was to search for pigmented cereals to propose as food or ingredients of food supplements combining their gluten free properties with the anti-inflammatory activity. To achieve this goal, the potential antioxidant and anti-inflammatory properties of extracts from pigmented rice in the framework of CD, and the mechanisms involved at cellular level, were evaluated.

## 2. Materials and Methods

### 2.1. Materials

Methanol, ethanol, HPLC-grade water, acetonitrile, acetone, toluene, reagents for electrophoretic analysis, and acids were from VWR International (Fontenay-sous-Bois, France). Gliadin, digestive enzymes, Folin-Ciocalteu’s reagent, 1,1-diphenyl-2-picryl-hydrazyl free radical (DPPH), salts, phenolic acids (ferulic acid, chlorogenic acid, vanillic acid, gallic acid, syringic acid, protocatechuic acid), rutin, and quercetin-3-*O*-glucoside were purchased from Sigma Aldrich (Steinheim, Germany). Anthocyanin standards were from Extrasynthese (Genay, France). Prestained molecular weight marker solution was from Bio-Rad (Hercules, CA, USA).

### 2.2. Rice Samples and Preparation of the Hydro-Alcoholic Extracts

Three pigmented brown rice varieties were included in the study. Violet rice was provided by “Azienda Agricola Bertolone Eleonora di Bertolone Giovanni”, Collobiano (VC); Venere and Nerone samples were purchased in the Italian market. Minute particles of the rice samples were obtained by grinding, using a coffee mill, and maintained at 4 °C until analysis. To evaluate the anti-inflammatory activity of pigmented rice at intestinal level, extracts were prepared according to Sangiovanni et al. [21], with small changes: 20 g of each rice flour was extracted twice with 200 mL of ethanol:water 60:40 (*v*/*v*) for 4 and 16 h, respectively, under dark conditions at room temperature. The hydro-alcoholic solution was used to optimize the extraction of polyphenols [22]. The mixtures obtained were filtered through filter paper, medium filtration rate, particle retention 10–20 μm (VWR, France), concentrated using a Rotavapor (Heidolph Instruments GmbH & CO, Schwabach, Germany), freeze-dried (Edwards, 5Pascal, Trezzano, Italy), and kept at −20 °C. Recovery (*w*/*w*) was 3.55% for Violet, 3.04% for Venere, and 3.79% for Nerone, respectively.

### 2.3. Peptic/Tryptic In Vitro Digestion

To reproduce a gliadin-related inflammatory state at the intestinal epithelium, the gliadin and the rice extracts were in vitro digested applying a peptic/tryptic protocol. The more complex gastrointestinal in vitro digestion reported by Sangiovanni et al. [21] and the peptic/tryptic protocol [13], applied on a hydro-alcoholic phenolic extract, similarly affected the phenolic compounds (data not shown); therefore the simplified protocol of Van Buiten et al. [13] was selected in this study to reduce interference on in vitro cellular assays.

The in vitro digestion was performed as described by Van Buiten et al. [13] with some adjustments; briefly, 2 g of gliadin or 0.1 g of rice extracts was suspended in 20 mL of 0.2 N HCl and stirred at 37 °C. After 10 min, pepsin (rate 1:50 enzyme:substrate *w*/*w*) was added to the suspension, and the solutions were stirred for 2 h at 37 °C. At the end of the gastric digestion, the pH was adjusted to 7.4 using 2 N NaOH, trypsin (rate 1:50 enzyme:substrate *w*/*w*) and the solutions were stirred for 4 h at 37 °C. At the end of the gastro-intestinal digestion, the solutions were freeze-dried and maintained at −20 °C until analysis. A blank, containing only the digestive enzymes, was prepared to evaluate possible interference.

The gliadin digestion was monitored using the Sodium Dodecyl Sulphate–PolyAcrylamide Gel Electrophoresis (SDS-PAGE) technique, in accordance with the method by Ballabio et al. [23]. Digested gliadin was suspended in sample buffer (containing 0.125 M Tris-HCl, pH 6.8, 3.75% glycerol, 1% SDS, 5% β-mercaptoethanol) diluted with water (1:1, *v*/*v*) on a polyacrylamide gradient gel (9–19% acrylamide). After the electrophoretic run (90 V at room temperature, for 6 h), the gels were maintained 20 min in 20% TCA at 4 °C and stained with Coomassie Brilliant Blue G-250.

### 2.4. Characterization of Phenolic Profile and Antioxidant Capacity of Rice Extracts

#### 2.4.1. Determination of Total Phenolic Content

The Total Phenolic Content (TPC) of extracts was determined according to the Folin–Ciocalteu assay [24]. Samples, both before and after digestion, were solubilized in ethanol:water 60:40 (*v*/*v*) at the final concentration of 2 mg extracts/mL and filtered using a 0.45 μm filter (VWR International, Fontenay-sous-Boys, France). A calibration curve was prepared using gallic acid standard solutions in the range of 5–50 μg/mL. The amount of 300 μL of diluted samples or standards was added with 1.5 mL of 0.2 N Folin–Ciocalteau reagent and 1.2 mL of 7.5% sodium carbonate. In parallel, the digestive blank or water were analyzed to remove interference. Solutions were maintained for 30 min in the dark and the absorbance was detected at 765 nm in a UV–visible spectrophotometer (Varian Cary 50 SCAN, Palo Alto, CA, USA). Results were expressed as mg/g equivalent of gallic acid (GAE).

#### 2.4.2. Determination of Total Anthocyanin Content

The total anthocyanin content (TAC) of rice extracts was determined applying the AOAC method [25]: samples, both before and after digestion, were solubilized in methanol: 1 M HCl (*v*/*v*) at the final concentration of 5 mg extracts/mL, and filtered using a 0.45 μm filter. Each sample solution was diluted with 0.025 M KCl (pH 1) and 0.4 M CH_3_COONa (pH 4.5). Detection of the absorbance was performed both at 520 nm and at 700 nm (to correct for haze).

The results were expressed as equivalents of cyanidin-3-*O*-glucoside (CY), in relation of the following equation:

TA (mg CY/g) = ∆A × MW × DF × 1000 × V/e × l × W,

where ∆A = (A520–A700 nm) pH 1.0–(A520–A700 nm) pH 4.5; MW: molecular weight (449.2 g/mol for CY); DF: dilution factor; 1000: conversion factor from g to mg; V: volume (L); e: molar extinction coefficient (26,900 for CY); l: path length in cm (1 cm); W: sample weight (g).

#### 2.4.3. Evaluation of the Antioxidant Capacity

The ability of the rice extracts to exert antioxidant capacity was evaluated spectrophotometrically applying the free radical 1,1-diphenyl-2-picryl-hydrazyl (DPPH) assay and the Trolox Equivalent Antioxidant Capacity (TEAC) test. Extracts, both before and after digestion, were solubilized in ethanol:water 60:40 (*v*/*v*) at the ultimate concentration of 2 mg extracts/mL and filtered using 0.45 μm filters.

##### DPPH Assay

In the DPPH assay [26], 1 mL of 0.005% DPPH in methanol was added to samples or blank (0.5 mL) and maintained in the dark for 30 min. The absorbance was measured against methanol at 517 nm. The antioxidant capacity was calculated using a calibration curve, built by plotting the concentration of gallic acid (1.0–5.0 μg/mL) versus the difference between the absorbance of the blank and the absorbance of standards (A0-A). The results were expressed as mg/g equivalents of gallic acid (GAE).

##### TEAC Assay

The Trolox Equivalent Antioxidant Capacity (TEAC) assay was carried out as described by Re et al. [27] with some adjustments: the solution of ABTS radical cation was prepared by mixing 2.45 mM of potassium persulfate with 7 mM ABTS (1:1 *v*/*v*); the obtained mixture was maintained at room temperature and in the dark for 12–16 h. The ABTS radical solution was diluted with ethanol before use to obtain an absorbance of 0.7 ± 0.02 at 734 nm. A total of 150 μL of each sample or blank was mixed with aliquots of 1.5 mL of ABTS+• solution and after 6 min the absorbance was measured at 734 nm.

The antioxidant capacity, expressed as mg/g of trolox equivalents (TE), was calculated using a calibration curve built plotting the concentration of trolox (10–30 μg/mL) versus the percentage of inhibition of ABTS+•, calculated as follows:

% Inhibition of ABTS+• = [(Ab − At)/Ab] × 100

where Ab: absorbance of blank; At: absorbance of test solutions.

#### 2.4.4. High-Performance Thin Layer Chromatography (HPTLC)

High-Performance Thin Layer Chromatography (HPTLC) is a technique useful to evaluate the samples’ fingerprint, testing in parallel their antioxidant properties.

The analyses were conducted as reported by Colombo et al. [28]. Briefly: 15 μL of rice extracts (8 mg extracts/mL ethanol:water 60:40 *v*/*v*) or blank of digestion and 5 μL of phenolic acids, rutin and quercetin-3-*O*-glucoside standard solutions (200 μg/mL) was loaded onto HPTLC silica-gel plates 60 F254 (10 × 20 cm, Merck, Darmstadt, Germany), making use of a semi-automatic sample applicator (Linomat 4, CAMAG, Muttenz, Switzerland). After the chromatographic run (mobile phase: 10 mL of acetone:toluene:formic acid 4.5:4.5:1 *v*/*v*/*v*), the plates were revealed at 366 nm, derivatized with 0.05% DPPH methanolic solution, maintained for 30 min in the dark and revealed at visible light (software VisionCats, CAMAG, Muttenz, Switzerland).

#### 2.4.5. High-Performance Liquid Chromatography-Diode Array Detector (HPLC-DAD) Analysis

The HPLC-DAD technique was applied for the quantitative analysis of anthocyanins and protocatechuic acid. The analyses were carried out using an HPLC equipment Jasco (Jasco, Tokyo, Japan) provided with an injection valve (Rheodyne, Cotati, CA, USA) with a 20 μL loop, a degasser (DG-2080-54), a mixer (LG-150-0.4), a diode array detector (MD-2010 Plus), an interface (LC-NETII/ADC), and a pump (PU-980).

The identification and quantification of anthocyanins were conducted applying the method reported by Colombo et al. [28], using a Synergi 4u MAX-RP 80A (250 × 4.60 mm 4 μm) (Phenomenex, Torrance, CA, USA) chromatographic column with a Security Guard C12 4 × 3.0 mm ID (Phenomenex, Torrance, CA, USA). The flow rate for the separation was set at 0.8 mL min^−1^. The gradient was set up as follows: 0–15 min: 94–70%, A, 15–30 min: 70–50% A, 30–35 min: 50–10% A, 35–38 min: 10% A isocratic and 38–48 min: 10–94% A; where (A) water:acetonitrile:formic acid 96:3:1 (*v*/*v*/*v*); and (B) acetonitrile:water:formic acid 50:49:1 (*v*/*v*/*v*). The anthocyanins were detected at 520 nm. Stock solutions of peonidin-3-*O*-glucoside (PE) and CY were prepared in methanol:1 M HCl 85:15 (*v*/*v*) obtaining a final concentration of 200 μg mL^−1^. The solutions were then suitably diluted in 0.1 N HCl at a concentration range of 0.5–20 μg mL^−1^ for CY and 0.5–10 μg mL^−1^ for PE.

The identification and quantification of protocatechuic acids were conducted using a chromatographic column Hibar^®^ 250–4.6, LiChrospher^®^ 100 RP-18 (250 × 4.60, 5 μm) (Merk, Darmstadt, Germany). The separation was performed at a flow rate of 0.8 mL min^−1^. The gradient was set up as follows: 0–10 min: 94–70%, A, 10–25 min: 70–30% A, 25–32 min: 30–10% A, 32–40 min: 10% A isocratic and 40–48 min: 10–94% A; where (A) water:acetonitrile:formic acid 96:3:1 (*v*/*v*/*v*); and (B) acetonitrile:water:formic acid 50:49:1 (*v*/*v*/*v*). The protocatechuic acid was detected at 280 nm. The standard solution was prepared in methanol:water 80:20 (*v*/*v*) obtaining a final concentration of 200 μg mL^−1^. The working solutions were then suitably diluted in water at the concentration range of 0.2–20 μg mL^−1^.

### 2.5. In Vitro Evaluation of the Anti-Inflammatory and Antioxidant Activity

#### 2.5.1. Cell Culture and Treatment

Caco-2 (clone HB237) from American tissue collection (ATCC, Manassas, VA, USA) cells was cultured in DMEM containing 100 mg streptomycin, 100 units penicillin per mL, 1% non-essential amino acids, 1 mM sodium pyruvate, 4 mM L-glutamine and 10% FBS (Fetal Bovine Serum) per mL. The cells were incubated under a humidified atmosphere with 5% CO_2_ at 37 °C until 50% of confluence; the medium was changed every other day. For the sub-culture biological test, cells were firstly detached from 75 cm^2^ flasks (Primo^®^; Euroclone, Pero, Italy) using Trypsin-EDTA 0.25% (GibcoTM; Thermo Fisher Scientific, Monza, Italy), counted, and placed in a new flask or seeded in plates (Falcon^®^; Corning Life Sciences, Amsterdam, The Netherlands).

For the treatments, Caco-2 cells were cultured for 48 h in 24 well plates (3 × 10^4^/well), then stimulated by the combination of IL-1β (10 ng/mL), IFN-γ (10 ng/mL), and in vitro digested gliadin (Ga) (1 mg/mL) pro-inflammatory cocktail. The read-out markers were evaluated after 6 h of stimulation and simultaneous treatment with rice extracts.

#### 2.5.2. Measurement of Cell Viability

The integrity of the cell morphology before and after each treatment was assessed by light microscope inspection. Cell viability was measured by the 3,4,5-dimethylthiazol- 2-yl-2-5-diphenylte-trazolium bromide (MTT) method as previously described [21]. In brief, MTT solution (Phosphate Buffer Solution, PBS 1X, 200 µg/mL) was added to cells after removing cell media at the end of each biological treatment. Then, cells were lysed by isopropanol:DMSO (90:10 *v*/*v*) solution after 15–30 min of MTT metabolization, to dissolve purple formazan salt. Finally, the absorbance was read at 550 nm and directly correlated with cell viability.

#### 2.5.3. Measurement of Soluble Inflammatory Markers by ELISA Assay

Human CXCL-8 (IL-8), CXCL-10, and sICAM-1 ELISA development ABTS kits were purchased from PeproTech (PeproTech, London, UK) and run according to the manufacturer’s instructions, as previously reported [29]. Corning 96-well EIA/RIA plates (Merck Life Science, Milano, Italy) were coated with the capture antibodies and kept at room temperature overnight. The amounts of inflammatory mediators in the culture media were detected by the measurement of the absorbance resulting from the colorimetric reaction between 2,20-azino-bis(3-ethylbenzothiazoline-6-sulfonic acid) (ABTS) substrate (Merck Life Science, Italy) and horseradish peroxidase enzyme, according to the manufacturer’s instructions. The signal was read using a spectrophotometer (VICTOR X3; PerkinElmer, Milano, Italy) at 405 nm. The obtained results (mean ± SD of at least three experiments) were expressed as percentage relative to the stimulated control, which was arbitrarily assigned the value of 100%. Apigenin (20 μM) was chosen as reference anti-inflammatory polyphenol, according to previously published papers concerning its bioactivity in chronic intestinal inflammation [30].

#### 2.5.4. Measurement of the NF-κB-Driven Transcription

NF-κB-driven transcription was evaluated as previously reported [21]. In brief, Caco-2 cells were transiently transfected with a reporter plasmid responsive to NF-κB (250 ng per well), containing the luciferase gene under the control of the E-selectin promoter characterized by three κB responsive elements. Lipofectamine^®^ 3000 Transfection Reagent (Invitrogen^®^; Thermo Fisher Scientific, Monza, Italy) was used for transfection assays. The plasmid was a gift from Dr. N. Marx (Department of Internal Medicine-Cardiology, University of Ulm; Ulm, Germany). BriteliteTM Plus reagent (Perkin Elmer, Milano, Italy) was used to assess the amount of luciferase produced into the cells, according to the manufacturer’s instructions. A VICTOR X3 Multilabel Plate Reader (Perkin Elmer, Milano, Italy) was used to measure the luminescence deriving from the reaction between luciferin and luciferase. The results (mean ± SEM of at least three experiments) were expressed as percentage relative to stimulated control, to which was arbitrarily assigned the value of 100%.

#### 2.5.5. Reactive Oxygen Species Analysis in Caco-2 Cells

Caco-2 cells were cultured in black 96-well plates for 5 days after seeding at a density of 20,000 cells/well; at 80% confluence the medium was removed, and the cells were treated with TNF-α (40 ng/mL) for 24 h as positive control, digested gliadin (500 μg/mL) and 200 μg/mL rice extracts alone or in combination with digested gliadin. At the end of the treatment, ROS were evaluated as previously described [31]: the medium was removed and 5 μM CellROX green in 50 μl DMEM W/O phenol red was added to each well. The cells were incubated for 30 min and then washed with PBS 3 times. Cells were lysed with 0.5% Tritonx-100. Plates were read by a spectrofluorometer (NIVO, Perkin Elmer) at an excitation of λ 485 and emission of λ 520. Results are expressed as Fluorescent Units (FI) [32].

### 2.6. K562(S) Cell Agglutination Test

The human erythroleukemic cell line K562(S) from ATCC, was maintained in RPMI-1640 (Roswell Park Memorial Institute) medium (Gibco, Carlsbad, CA, USA) supplemented with 10% fetal calf serum (Gibco), 1% 100× penicillin streptomycin and subcultured every 3 days to maintain a cell density of 10 × 105/mL.

Cells were harvested and washed twice by centrifugation with Ca^2+^ and Mg^2+^-free PBS (Gibco) for the agglutination experiments [33]. The test was conducted with resuspended cells at a concentration of 10 × 10^7^ cells/mL in the same PBS. Briefly, 25 µL of cell suspension was additioned to each well of a 96-well microtiter plate containing digested gliadin (0.75–3 mg/mL) or zein digest (6 mg/mL) concentrations obtained by serial dilution (1:1) with PBS, and then 25 μL PBS was added in each well. The inhibition test was carried out by adding rice hydro-alcoholic extracts (3 mg/mL) instead of 25 μL PBS before the addition of cells to the digested gliadin wells.

The obtained total volume was 100 µL. The cell suspension was incubated at room temperature for 30 min.

Clumped and single cells were counted on the microscope slide after the application of a drop of suspension. Control cells were included, as appropriate. Unless otherwise specified, the agglutination test was repeated twice [33].

### 2.7. Statistical Analysis

Comparison of analytical data between extracts before and after digestion was performed by parametric paired t-test using GraphPad Prism 9 software (GraphPad Software Inc., San Diego, CA, USA). The correlation between variables was assessed by Pearson linear correlation using IBM SPSS Statistics, Version 27.0 (New York, NY, USA). All biological data were expressed as the mean ± SEM of at least three independent experiments. Unpaired one-way analysis of variance (ANOVA) was used to analyze the quantitative assays, followed by the Bonferroni post-hoc test using GraphPad Prism. Values of *p* < 0.05 were considered statistically significant.

## 3. Results and Discussion

### 3.1. Characterization of Rice Extracts

#### 3.1.1. Evaluation of Phenolic Profile and Antioxidant Capacity

The rice extracts were chemically characterized applying different spectrophotometric methods. Table 1 shows the Total Phenolic Content (TPC) and the Total Anthocyanin Content (TAC) measured in samples, both before and after in vitro digestion. In parallel, the Antioxidant Capacity (AOA) was determined through DPPH and TEAC assays.

The TAC in extracts ranged between 32.6 ± 0.4 and 66.3 ± 1.5 mg CY/g for Violet and Nerone, respectively. Considering the yield of extraction, these data corresponded to 2.5 ± 0.1 mg CY/g flour in Nerone rice, 1.2 ± 0.01 mg CY/g flour in Violet rice, and 1.0 ± 0.1 mg CY/g flour in the Venere variety. Nerone showed a significantly higher content of total anthocyanins (*p* < 0.05), while not statistical differences were observed between the Violet and Venere flour (*p* < 0.05). These data agree with Min et al. who reported a total anthocyanin content of 4.13 mg CY/g flour (d.w.) and 1.17 mg CY/g flour in two purple rice varieties [34]. The TAC statistically decreased in all samples after the gastrointestinal digestion (*p* < 0.01). The reduction was higher for Nerone (−56%) compared to Violet and Venere (−37.7% and −37.1%, respectively). Anthocyanins are stable under gastric conditions [35,36], while the pH of the intestine, close to neutrality, converts anthocyanins into several metabolites, including phenolic acids and aldehydes [37]. Our data agree, although at a slightly lower level, with Sun et al., who observed a reduction of 76% of total anthocyanins after the in vitro gastrointestinal digestion of a purple rice extract [38].

The TPC ranged between 124.1 ± 4.2 and 159.9 ± 16.7 for Violet and Nerone, respectively. Considering the yield of extraction, these data corresponded to 6.1 ± 0.6 mg GAE/g flour, 4.4 ± 0.1 mg GAE/g flour, and 4.1 ± 0.1 mg GAE/g flour for Nerone, Violet, and Venere, respectively. Nerone showed the highest total phenolic content (*p* < 0.05) compared to the other extracts. These results agree with data reported by Min et al., who observed a SPC from 0.30 to 7.03 mg GAE/g flour (d.m.) in six rice varieties with different bran color (from white to purple); the pigmented varieties (red and purple) showed the highest content of polyphenols [34]. Melini et al. found slightly higher data in four black rice varieties (6.79–15.08 mg GAE/g d.m.) [39]. No statistical differences (*p* > 0.05) were observed in TPC after the in vitro gastro-intestinal digestion of the Nerone and Violet samples, while a reduction of 27.9% was observed in the Violet extract (*p* < 0.01).

The in vitro digestion impacted on the AOA of the Violet and Venere extracts; the AOA of Violet rice was the most affected by the in vitro digestion with a significant reduction of 35% and 29%, evaluated through DPPH and TEAC, respectively. It is interesting to note that the antioxidant capacity of Nerone, measured with both DPPH and TEAC assays, was slightly higher after gastrointestinal digestion (+13.7%, +14.5%, *p* < 0.05). The TPC significantly correlates with AOA, measured both with DPPH (r = 0.729, *p* < 0.001) and TEAC (r = 0.952, *p* < 0.001). No significant correlation was observed between TAC and AOA (*p* > 0.01), suggesting that other molecules, after in vitro digestion, contribute to the AOA of samples.

Different studies have reported the contrasting effects of digestion processes on phenolic content in different matrices, showing both a decrease [40,41] and an increase [42,43,44].

The increase in the TPC or AOA of samples could be explained by the transformation of phenolics in compounds characterized by a higher antioxidant capacity [44] or by the action of digestive enzymes that can break chemical bonds in the phenolics and proteins, determining the release of phenolics, which represent major constituents of the cell wall [42]. In agreement with our data, in vitro digestion can differently impact different samples: Wootton-Beard et al. determined the total antioxidant capacity of 23 vegetable juices before and after an in vitro simulated digestion. After the gastric phase, a significant (*p* < 0.05) increase in total polyphenol content was observed for all the juices considered. After the duodenal phase of digestion, 88.6% of the juices showed a further increase in total polyphenol content [43]. The antioxidant capacity of the samples remained high throughout the in vitro digestion. As observed for Violet rice, Peanparkdee et al. determined a reduction of TPC, FRAP (Ferric Reducing Antioxidant Power Assay), and DPPH values in extracts from pigmented and non-pigmented rice bran after gastrointestinal digestion [40].

#### 3.1.2. Identification and Quantification of Phenolic Compounds Applying Chromatographic Techniques

**Figure 1:**
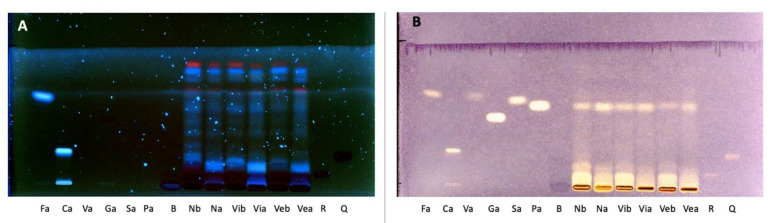
HPTLC plate of standards and samples detected at 366 nm (**A**) and at visible light after derivatization with DPPH (**B**). Fa: Ferulic acid, Ca: Chlorogenic acid, Va: Vanillic acid, Ga: Gallic acid, Sa: Syringic acid, Pa: Protocatechuic acid, B: Blank of digestion, Nb: Nerone before digestion, Na: Nerone after digestion, Vib: Violet before digestion, Via: Violet after digestion, Veb: Venere before digestion, Vea: Venere after digestion, R: Rutin, Q: Quercetin-3-*O*-glucoside.

To study the differences between rice samples during gastrointestinal digestion, the High-Performance Thin Layer Chromatography (HPTLC) technique was applied. High-Performance Thin Layer Chromatography is a semi-quantitative, fast and suitable method for the evaluation of the phenolic profile of samples. In addition, the HPTLC technique can be used to evaluate the antioxidant activity associated with each compound present in the samples. Figure 1 shows the HPTLC separation of samples before and after in vitro digestion. The antioxidant capacity of samples was evaluated using DPPH as the derivatization agent: the discoloration of the spots (due to DPPH reduction) from violet to yellow is proportional to their AOA.

The phenolic profile of all samples changed after in vitro digestion (Figure 1A). Notably, the band corresponding to protocatechuic acid increased after digestion in all extracts, particularly in Nerone rice (Figure 1B), and it was also characterized by a high antioxidant capacity. Indeed, this HPTLC method is useful to evaluate the antioxidant capacity of specific compounds, in contrast to spectrophotometric methods that provide information about the total antioxidant capacity.

Subsequently, the HPTLC-DAD technique allowed: (1) the identification and quantification of the anthocyanins and their modifications after in vitro digestion; (2) the quantification of the protocatechuic acid released during gastro-intestinal digestion.

**Figure 2:**
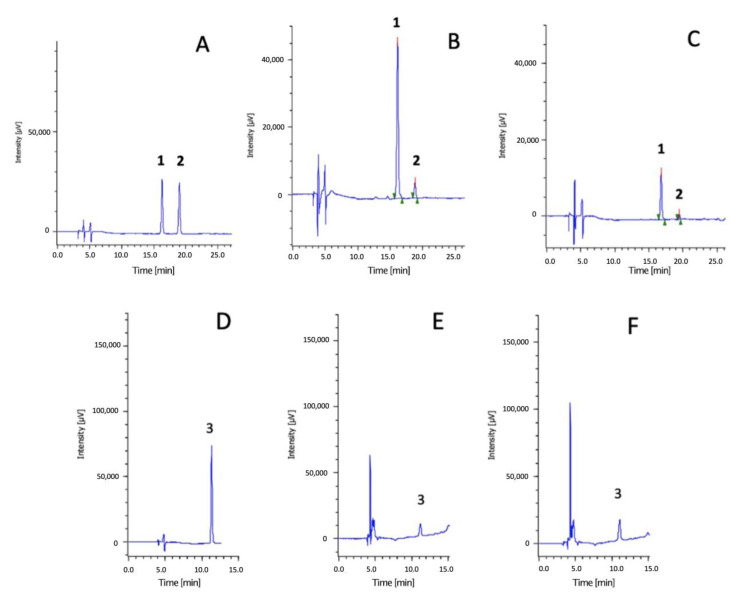
Chromatograms of (**A**) anthocyanin standard solution (5 μg/mL); (**B**) Nerone extract before digestion, detection at 520 nm; (**C**) Nerone extract after digestion, detection at 520 nm; (**D**) protocatechuic acid (20 μg/mL); (**E**) Nerone extract before digestion, detection at 280 nm; (**F**) Nerone extract after digestion, detection at 280 nm; (1) cyanidin-3-*O*-glucoside, (2) peonidin-3-*O*-glucoside, (3) protocatechuic acid.

Figure 2 shows the chromatograms obtained with anthocyanin standard solution (5 μg/mL), protocatechuic acid standard solution (20 μg/mL), and Nerone rice before and after digestion. In addition, Table 2 lists the identification and quantification of anthocyanins and protocatechuic acid by the HPLC-DAD method.

The HPLC-DAD technique showed that cyanidin-3-*O*-glucoside was the most representative anthocyanin in rice, followed by peonidin-3-*O*-glucoside [34,45]. The total anthocyanin content ranged between 47.2 ± 1.0 and 20.3 ± 0.1 mg/g extract for Nerone and Venere, respectively. Considering the yield of extraction, these data correspond to 1.79 ± 0.04 mg/g flour, 0.82 ± 0.03 mg/g flour, and 0.62 ± 0.03 mg/g flour for Nerone, Violet, and Venere, respectively. These results agree with the literature data: studying thirty pigmented varieties of rice, Bhuvaneswari et al. reported an anthocyanin content ranging from 0.30 to 2.76 mg/g [45]. According to the spectrophotometric results, the anthocyanins significantly decreased during in vitro digestion (*p* < 0.001); in parallel, a significant increase in protocatechuic acid (*p* < 0.001) was observed in all samples. This was particularly evident in the Nerone extract followed by Venere rice, according to the stability of the AOA observed in the spectrophotometric assays.

The most representative anthocyanin of rice samples was cyanidin-3-*O*-glucoside (CY). Different studies reported that the most abundant metabolite of CY was protocatechuic acid [46,47]. Protocatechuic acid is one of the phenolic acids present in rice with the highest antioxidant activity [48]. The degradation of anthocyanins into smaller molecules, such as benzoic acid derivatives, could explain the stability of total phenolic compounds and the slightly increment of AOA after digestive processes observed in Nerone rice, the sample in which was observed the highest increase in protocatechuic acid (+103%).

### 3.2. In Vitro Evaluation of the Biological Activity of Extracts

#### 3.2.1. Effect of Rice Extracts on Inflammatory Markers in Caco-2 Cells

To address the potential role of rice extracts in CD-related intestinal inflammation, we firstly reproduced a pro-inflammatory milieu for in vitro treatment by combining leukocyte-derived cytokines (IL-1β, IFN-γ, 10 ng/mL) and previously digested gliadin (Ga, 1 mg/mL) at physiologically relevant concentrations. Gliadin was extensively in vitro-digested (Figure S1). The pathogenesis of CD starts with the incomplete digestion of gluten, which triggers an increase in intestinal permeability [49]. The gliadin peptides in celiac subjects can activate the innate and adaptive immune response with the production of proinflammatory cytokines and auto-antibodies. The in vitro digestion of gliadin is therefore important to develop an in vitro model able to mimic a gliadin-related inflammatory state at gut level.

IFN-γ is known to enhance the inflammatory response to innate cytokines in epithelial cells, including Caco-2 [4], but the role of gliadin has been poorly investigated. According to our experience and that of other authors, Caco-2 cells are mainly responsive to IL-1β [21,50,51]; for this reason, we evaluated the impacts of IFN-γ and Ga on IL-1β stimulation by measuring the release of several inflammatory markers (Figure S2). As expected, IL-1β challenge was necessary to induce IL-8, CXCL-10, and sICAM-1 release; IFN-γ enhanced the IL-1β-induced release of all the markers tested, although the effect on IL-8 was not statistically significant. The addition of Ga to IL-1β/IFN-γ caused the further significant enhancement of IL-8 and CXCL-10 release, but not sICAM-1.

**Figure 3:**
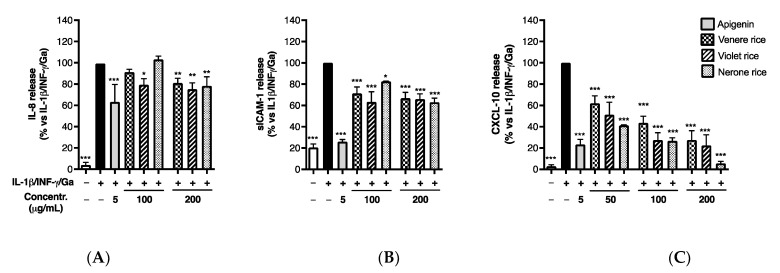
Effect of black rice extracts on IL-8 (**A**), sICAM-1 (**B**), and CXCL-10 (**C**) release. Caco-2 cells were stimulated by IL-1β (10 ng/mL), IFN-γ (10 ng/mL), and in vitro digested gliadin (Ga) (1 mg/mL) for 6 h. Unstimulated condition was also reported (white bar, Control −). The release of inflammatory mediators in cell culture media was measured by ELISA assay and expressed as mean of release (% ± SEM) vs. stimulus (black bar, Control+), to which was arbitrarily attributed the value of 100%. * *p* < 0.05, ** *p* < 0.01, *** *p* < 0.001 vs. stimulus.

Therefore, hydro-alcoholic extracts from the three selected black rice varieties (Venere, Violet, Nerone) were tested in Caco-2 cells, challenged by IL-1β/IFN-γ/Ga pro-inflammatory mix, at physiologically relevant concentrations after hypothetical oral consumption (50–200 μg/mL). All three extracts showed no cytotoxicity even at the highest concentration tested (200 μg/mL) at the end of the treatments (24 h). They significantly impaired the release of sICAM-1, IL-8, and CXCL-10 in a comparable fashion (Figure 3); however, the inhibitory effect, ranging from 34% to 37% for sICAM-1 and from 20% to 25% for IL-8 at the highest concentration tested (200 μg/mL), was more pronounced on CXCL-10 release, as evident from the IC_50_s reported in Table 3. In line with previous results, indicating a higher content of total phenolics (see Table 1), the extract from the Nerone variety emerged for the lower IC_50_, corresponding to 21.53 μg/mL (a wider concentration–response curve is reported in Figure S3A).

**Figure 4:**
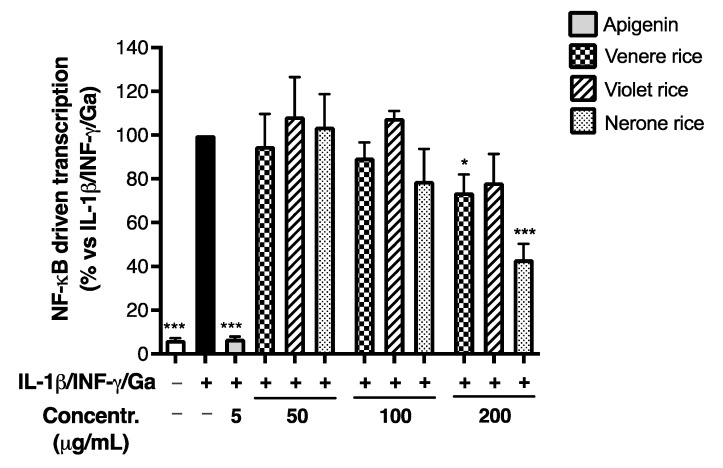
Effect of black rice extracts on NF-κB-driven transcription. Caco-2 cells were stimulated by IL-1β (10 ng/mL), IFN-γ (10 ng/mL), and in vitro digested gliadin (Ga) (1 mg/mL) for 6 h. Unstimulated condition was also reported (white bar, Control −). The NF-κB-driven transcription was measured by luciferase assay and expressed as mean emission ± SEM vs. stimulus (black bar, Control +), to which was arbitrarily attributed the value of 100%. * *p* < 0.05, *** *p* < 0.001 vs. stimulus.

Since NF-κB plays a key role in the transcription of chemokines and adhesion molecules during inflammatory processes, we evaluated the effect of black rice extracts on the NF-κB-driven transcription using the same pro-inflammatory cocktail as previously: once again, the Nerone extract exhibited the highest inhibitory effect, with an IC_50_ of 165.90 ± 33.75 μg/mL (Figure 4), while the Venere and Violet extracts showed only a slight inhibitory activity at the highest concentration tested (200 μg/mL). Despite the inhibitory concentrations higher than those required for CXCL-10 impairment, the experiment suggested a plausible mechanism involved in the biological activity previously observed.

To better support the relevance of NF-κB activation in our in vitro stimulatory settings, we measured the impact of each component of the inflammatory mix on the NF-κB-driven transcription (Figure S4). Differently from other authors, we observed that Ga (1 mg/mL) was unable to trigger the NF-κB pathway, suggesting that gliadin might directly activate it [10]. The same was observed after the stimulation with IFN-γ (10 ng/mL) alone. On the contrary, IL-1β (10 ng/mL) increased the transcriptional activity, as expected, but the combination with the other stimuli did not cause an additional increase. In comparison with the methodological conditions shown in Figure S2, these findings attribute to IL-1β/NF-κB signaling the main relevance in our pro-inflammatory setting. Consequently, we supposed IL-1β uniquely responsible for the NF-κB activation, while Ga and IFN-γ could have contributed to the production of inflammatory mediators through other signals, such as the STAT pathway and oxidative stress.

**Figure 5:**
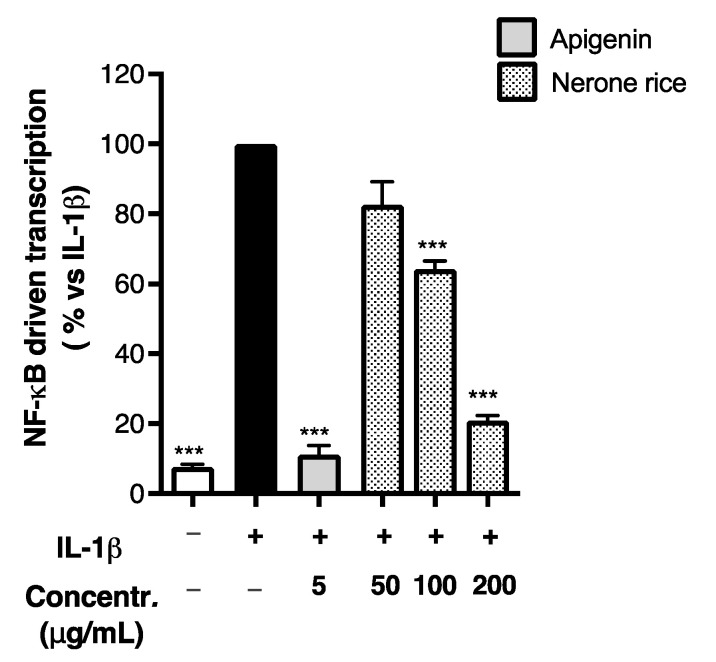
Effect of black rice extract from Nerone variety on the NF-κB-driven transcription. Caco-2 cells were stimulated by IL-1β (10 ng/mL) for 6 h. Unstimulated condition was also reported (white bar, Control −). The NF-κB-driven transcription was measured by luciferase assay and expressed as mean ± SEM vs. stimulus (black bar, Control +), to which was arbitrarily attributed the value of 100%. *** *p* < 0.001 vs. stimulus.

For this reason, we repeated the cell treatment with the Nerone extract using IL-1β stimulation alone. As expected, the Nerone extract impaired the NF-κB-driven transcription with an IC_50_ of 108.4 ± 9.57 μg/mL (Figure 5), thus strengthening the idea that this molecular mechanism is involved in its inhibitory activity.

#### 3.2.2. Impact of In Vitro Simulated Digestion on Anti-Inflammatory Activity

**Figure 6:**
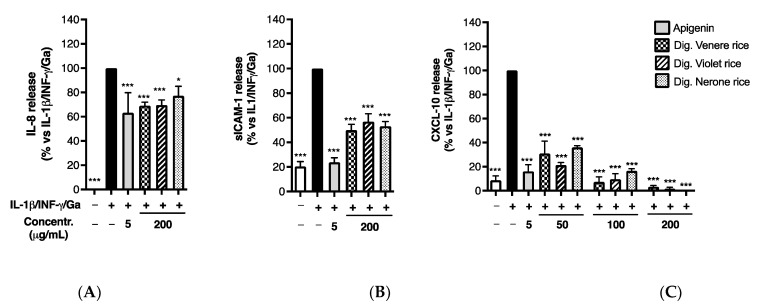
Effect of black rice extracts on IL-8 (**A**), sICAM-1 (**B**), and CXCL-10 release (**C**) after in vitro simulated digestion. Caco-2 cells were stimulated by IL-1β (10 ng/mL), IFN-γ (10 ng/mL), and in vitro digested gliadin (Ga) (1 mg/mL) for 6 h. Unstimulated condition was also reported (white bar, Control −). The release of inflammatory mediators in cell culture media was measured by ELISA assay and expressed as mean of release (% ± SEM) vs. stimulus (black bar, Control +). To the stimulus was arbitrarily attributed the value of 100%. * *p* < 0.05, *** *p* < 0.001 vs. stimulus.

We previously described the impact of the in vitro simulated digestion on the TPC and AOA of the black rice extracts (Table 1 and Table 2). Accordingly, we evaluated the impact on biological activity; all the extracts showed a comparable inhibitory effect on IL-8 and sICAM-1 release at the highest concentration tested before the digestive simulation (200 μg/mL) (Figure 6A,B). This evidence suggested a negligible impact of the in vitro digestion on bioactivity, although a reduction in the TPC was generally observed.

Similarly, all black rice extracts maintained their inhibitory activity on CXCL-10 release, with IC_50_s below to 50 μg/mL (Figure 6C), while the bioactivity of the Venere and Violet extracts showed a tendency to increase in comparison to the previously calculated IC_50_s (Table 3). Of note, the bioactivity of the Nerone extract exhibited a slight decrease, since the IC_50_ rose from 21.53 ± 7.29 to 31.38 ± 4.12 μg/mL (a wider concentration–response curve is reported in Figure S3B). However, the difference was not statistically significant. A possible explanation may concern the common increase in phenolic acids after in vitro digestion. As evident from HPTLC and HPLC analysis (see Figure 1 and Table 1), the phenolic acid PA significantly increased after digestion in all the extracts, whereas a stronger reduction in the TAC for the Nerone extract was observed (Table 1).

**Figure 7:**
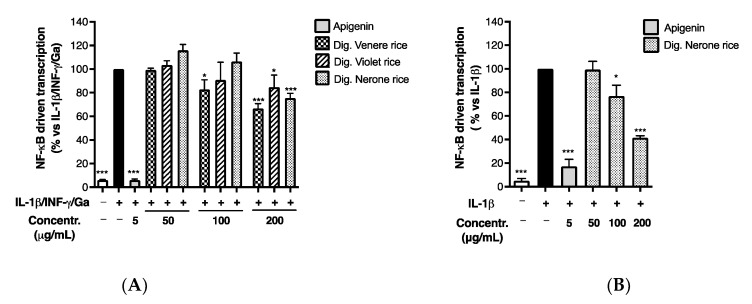
Effect of black rice extract from Nerone variety on NF-κB-driven transcription after in vitro simulated digestion. Caco-2 cells were stimulated by IL-1β (10 ng/mL) for 6 h. Unstimulated condition was also reported (white bar, Control −) (**A**). The NF-κB-driven transcription was measured by luciferase assay and expressed as mean emission ± SEM vs. stimulus (black bar, Control +), to which was arbitrarily attributed the value of 100% (**B**). * *p* < 0.05, *** *p* < 0.001 vs. stimulus.

The data paralleled the bioactivity on the NF-κB-driven transcription, since the Venere and Violet extracts exhibited a slightly higher inhibitory effect, while the Nerone extract displayed a slightly lower activity after in vitro simulated digestion (Figure 7A). Again, the latter observation was also confirmed during IL-1β challenge (Figure 7B), since the IC_50_ on NF-κB-driven transcription moderately increased from 108.4 ± 9.57 to 164 ± 14.10 μg/mL (Table 4).

#### 3.2.3. In Vitro Antioxidant Activity of Rice Extracts

**Figure 8:**
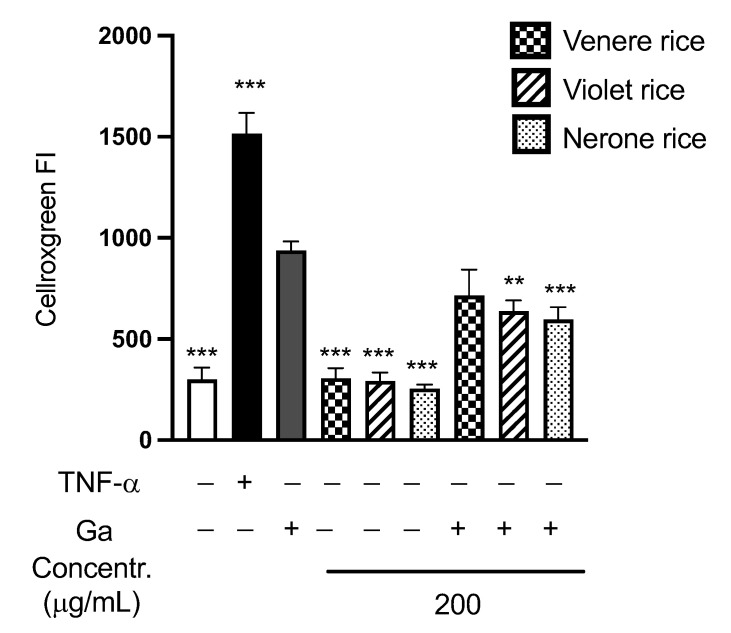
Intracellular reactive oxygen species (ROS) levels in Caco-2 cells, measured as the fluorescence intensity (FI) of Cellrox Green. Caco-2 cells were treated for 24 h with 500 μg/mL digested gliadin (Ga) or rice extracts (200 μg/mL). A set of samples were exposed to rice extracts 3 h before exposure to Ga. Levels of ROS in the untreated cells (white bar, Control −) and in cells treated only with TNF-α 40 ng/mL (black bar, Control +) were also reported. Data are the means (± SD) of three replicates analyzed in duplicate. ** *p* < 0.01, *** *p* < 0.001 vs. Ga stimulus 500 μg/mL.

In celiac patients, gliadin is involved in the production of Reactive Oxygen Species (ROS) at the intestinal level [2]. The ability of rice extracts (at 200 μg/mL) to reduce the ROS level in Caco-2 cells after the stimulation with digested gliadin was evaluated (Figure 8).

As expected, digested gliadin (Ga) induced the production of ROS compared to the negative control. Treating cells for 3 h before the exposure to Ga reduced the production of ROS compared to the cells treated with Ga alone and this reduction was significant for Nerone (*p* < 0.001) and Violet (*p* < 0.05). These data confirmed the antioxidant activity of all the rice extracts in Caco-2 cells.

#### 3.2.4. K562(S) Cell Agglutination Test

**Figure 9:**
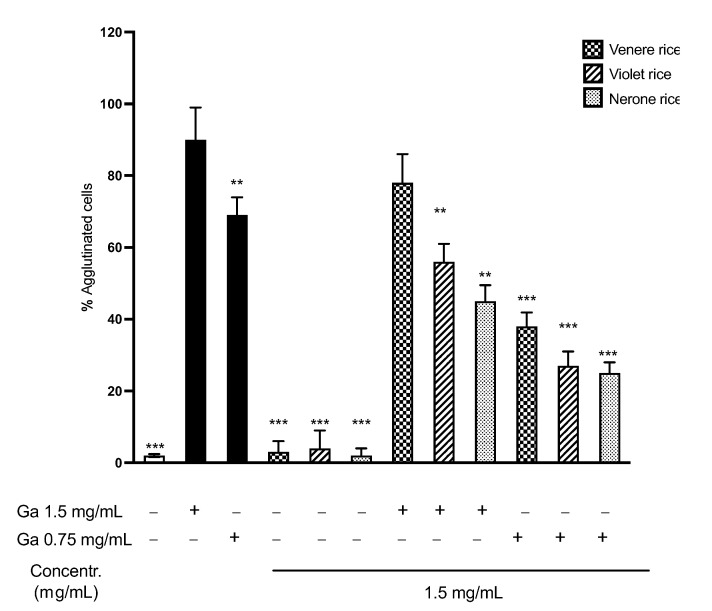
Agglutination test on K562(S) cells. Cells were incubated for 30 min in the presence of digested gliadin (Ga) at different concentrations (1.5 and 0.75 mg/mL) and rice extracts at 1.5 mg/mL. Unstimulated condition was also reported (white bar). A set of sample wells were incubated 30 min before with rice extracts and then incubated for another 30 min with Ga both at 1.5 mg/mL and 0.75 mg/mL. Results are expressed as % of agglutinated cells. ** *p* < 0.01, *** *p* < 0.001 vs. Ga stimulus 1.5 mg/mL.

In myelogenic leukemia cells K562(S), the toxic gliadin peptides activate trasglutaminase-2, a key enzyme involved in the pathogenesis of CD, determining the cytoskeleton rearrangement and, consequently, the cell agglutination. The K562(S) cell line represents a useful and rapid tool for evaluating the activation of epithelial cells in CD and their modulation induced by rice extracts. The cells were incubated with digested gliadin (Ga) and/or rice extracts (Figure 9).

As expected, Ga induced a massive, concentration-dependent agglutination of cells that was not observed treating cells with rice extracts alone. Rather, pre-treating cells with extracts before the addition of Ga determined a significant reduction in the % of agglutination, particularly for Nerone rice, according to the high phenolic content of this extract.

## 4. Conclusions

The present work suggests a potential anti-inflammatory and antioxidant effect of black rice at the intestinal level, with a focus on celiac disease. Our experiments demonstrated the possible impact of rice extracts at concentrations that could be reached within a regular diet (50 μg/mL) on the production of reactive oxygen species and inflammatory mediators from intestinal epithelial cells, with particular attention to lymphocyte-attracting CXCL-10. From the analytical results, and according to the literature [29,52], we suppose that the presence of anthocyanins and their digestion metabolites (such as protocatechuic acid) may account for the observed anti-inflammatory activity after in vitro simulated digestion, probably through the impairment of the NF-κB pathway. However, further molecular mechanisms related to the IFN-γ pathway and gliadin-induced cellular damage could be involved in the release of inflammatory cytokines.

In addition, the data obtained in K562(S) opened further insight regarding the possible role of phenolic-rich extracts in modulating the early activation of epithelial cells induced by gliadin. Notably, the Nerone extract contained the highest quantity of anthocyanins and PA, which correlated with the most interesting effects of agglutination reduction, NF-κB, and CXCL-10 release. Moreover, the main anthocyanin from the Nerone extract, namely cyanidin-3-*O*-glucoside, demonstrated anti-inflammatory properties in models of intestinal inflammation [53].

Within our perspectives, this work should represent a solid base for future investigations that will provide data for the potential use of black rice as food supplements or a healthy food for celiacs. From a nutritional point of view, our evidence could be sustained by dietary intervention studies aimed at elucidating the potential role of cooked black rice, with all its associated variables, or black rice-derived extracts.

## Figures and Tables

**Table 1:** Characterization of rice extracts in terms of Total Phenolic Content (TPC), Total Anthocyanin Content (TAC), and Antioxidant Capacity (AOA). Results (mean ± SD, *n* = 3) are expressed as mg equivalent of gallic acid (GAE)/g, mg equivalent of cyanidin-3-*O*-glucoside (CY)/g or mg trolox equivalent (TE)/g.

Samples	Digestion	TAC mg CY/g	TPC mg GAE/g	AOA	
				DPPH mg GAE/g	TEAC mg TE/g
Nerone	Before	66.26 ± 1.54	159.92 ± 16.68	40.97 ± 0.85	301.71 ± 6.50
	After	29.23 ± 2.80 **	175.87 ± 4.94	46.60 ± 0.32 *	345.37 ± 23.04 *
Violet	Before	32.61 ± 0.41	124.09 ± 4.22	45.52 ± 0.49	240.54 ± 18.72
	After	20.31 ± 1.37 **	89.53 ± 6.51 **	29.39 ± 2.26 **	170.35 ± 8.94 *
Venere	Before	34.28 ± 2.67	135.58 ± 2.11	43.19 ± 3.68	267.23 ± 11.48
	After	21.57 ± 1.49 **	120.61 ± 10.99	38.27 ± 3.50	233.40 ± 4.84 *

* *p* < 0.05, ** *p* < 0.01.

**Table 2:** Identification and quantification of anthocyanins and protocatechuic acid by the HPLC-DAD technique. Results are expressed as mean ± SD (*n* = 3).

Samples	Digestion	Anthocyanins (mg/g)				Phenolic Acids (mg/g)	
		CY	PE	Total (CY + PE)	% Variation	PA	% Variation
Nerone	Before	43.440 ± 0.944	3.724 ± 0.048	47.165 ± 0.969		1.546 ± 0.016	
	After	11.384 ± 0.136	1.187 ± 0.010	12.571 ± 0.126 ***	−73.3	3.140 ± 0.032 ***	+103.0
Violet	Before	22.334 ± 0.928	0.786 ± 0.024	23.120 ± 0.928		2.056 ± 0.097	
	After	6.258 ± 0.058	0.212 ± 0.002	6.470 ± 0.057 ***	−72.0	3.017 ± 0.056 ***	+46.75
Venere	Before	19.242 ± 0.090	1.012 ± 0.007	20.254 ± 0.091		1.823 ± 0.010	
	After	4.723 ± 0.046	0.205 ± 0.001	4.928 ± 0.047 ***	−75.7	2.769 ± 0.037 ***	+51.91

CY: cyanidin-3-*O*-glucoside; PE: peonidin-3-*O*-glucoside; PA: protocatechuic acid; *** *p* < 0.001.

**Table 3:** IC_50_s of extracts from black rice varieties (Ve, Vi, Ne) on CXCL-10 release.

Black Rice Variety	IC_50_ (μg/mL)
Ve	76.44 ± 13.59 ***
Vi	48.07 ± 13.02 ***
N	21.53 ± 7.29 ***

Ve, Venere rice; Vi, Violet rice; N, Nerone rice; *** *p* < 0.001.

**Table 4:** IC_50_s of extracts from Nerone on the NF-κB-driven transcription induced by IL-1β/IFN-γ/Ga pro-inflammatory mix or IL-1β alone.

Nerone Extract	IC_50_ (μg/mL) vs. IL-1β/IFN-γ/Ga	IC_50_ (μg/mL) vs. IL-1β
Before digestion	165.90 ± 33.75	108.4 ± 9.57
After digestion	>200 μg/mL	164 ± 14.10

## Data Availability

All experimental data are included in the text of the paper or in the Supplementary Materials.

## Supplementary Materials

The following supporting information can be downloaded at: https://www.mdpi.com/article/10.3390/foods12010063/s1, Figure S1: electrophoretic pattern of gliadin before and after in vitro digestion; Figure S2: enhancing effect of IFN-γ and gliadin on the release of inflammatory mediators; Figure S3: concentration–response effect of Nerone rice extract on CXCL-10 release before and after in vitro digestion; Figure S4: IFN-γ and gliadin and their ability to enhance NF-κB-driven transcription.
