# Supplementary material for: Evaluation of the Potential Anti-Inflammatory Activity of Black Rice in the Framework of Celiac Disease
Source: Foods. 2022 Dec 22;12(1):63. doi: 10.3390/foods12010063 (PMC9818972; doi:10.3390/foods12010063)
Supplement: Supplementary file 1 [file foods-12-00063-s001.zip › foods-2026666-supplementary.pdf]

Supplementary

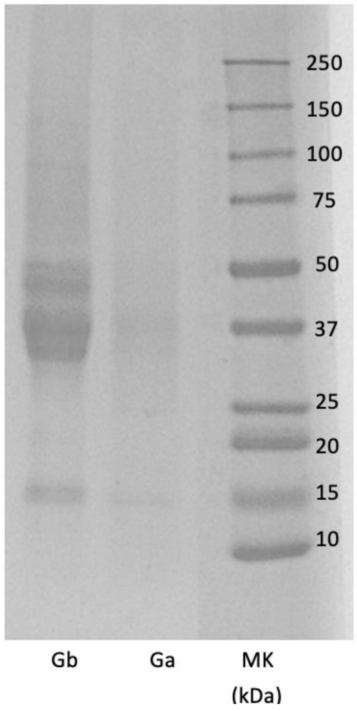

Figure S1. Electrophoretic pattern of gliadin before (Gb) and after (Ga) in vitro digestion. MK: molecular weight marker solution

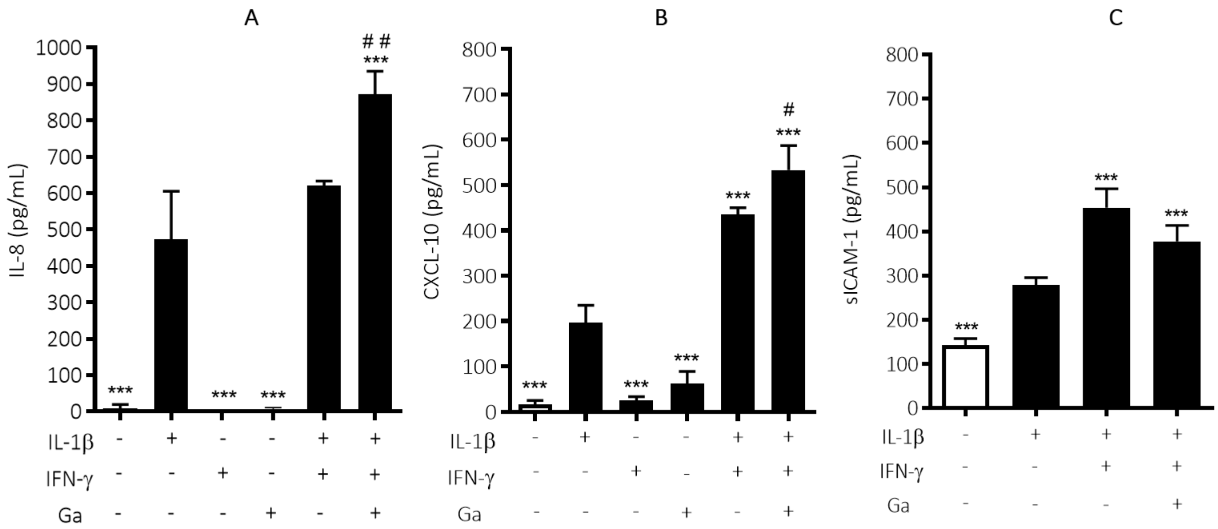

Figure S2. Enhancing effect of IFN-γ and gliadin on the release of inflammatory mediators. Caco-2 cells were stimulated by IL-1β (10 ng/mL), IFN-γ (10 ng/mL), and *in vitro* digested gliadin (Ga) (1 mg/mL) for 6h. The release of inflammatory mediators in culture media was measured by ELISA

assay and expressed as mean pg/mL of protein release ± SEM. ***p <0.001 vs IL-1β, #p < 0.05 vs IL-1β/IFN-γ.

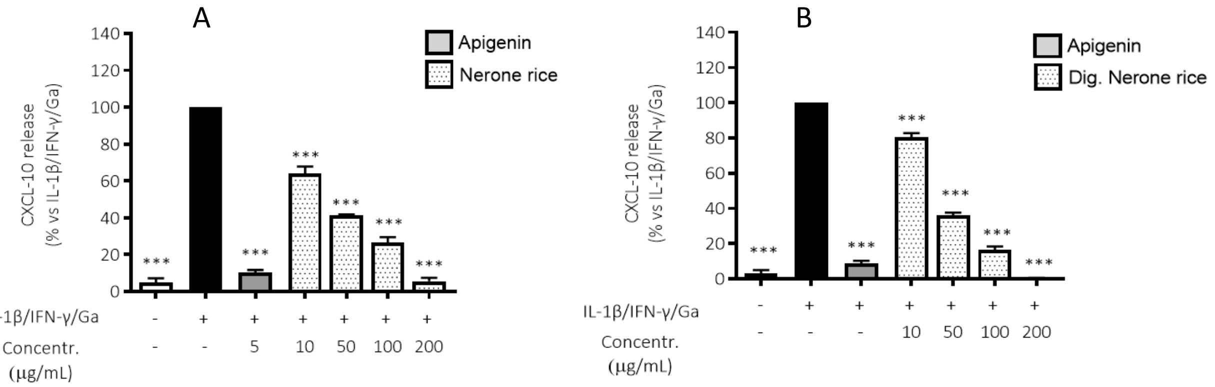

Figure S3. Concentration-response effect of Nerone rice extract on CXCL-10 release before (A) and after (B) *in vitro* simulated digestion. Caco-2 cells were stimulated by IL-1β (10 ng/mL), IFN-γ (10 ng/mL), and *in vitro* digested gliadin (Ga) (1 mg/mL) for 6h. The release of CXCL-10 in culture media was measured by ELISA assay and expressed as mean of release (% ± SEM) vs stimulus (black bar, Control +), to which was arbitrarily attributed the value of 100%. *p < 0.05, **p < 0.01, ***p < 0.001 vs CTRL.

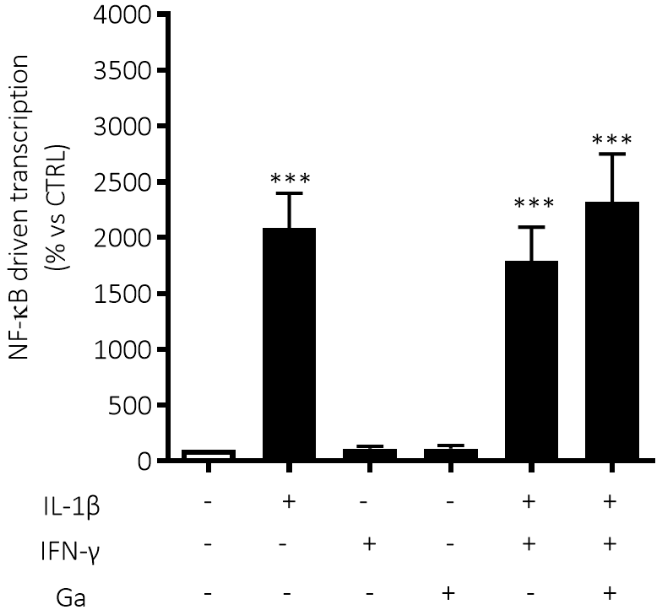

Figure S4. IFN-γ and gliadin (Ga) are unable to enhance NF-κB-driven transcription. Caco-2 cells were stimulated by IL-1β (10 ng/mL), IFN-γ (10 ng/mL), and *in vitro* digested gliadin (Ga) (1 mg/mL) for 6h. The NF-κB-driven transcription was measured by luciferase assay and expressed as mean emission ± SEM. ***p < 0.001 vs CTRL.
